# Crystal Structure of *Bacillus subtilis* Cysteine Desulfurase SufS and Its Dynamic Interaction with Frataxin and Scaffold Protein SufU

**DOI:** 10.1371/journal.pone.0158749

**Published:** 2016-07-06

**Authors:** Bastian Blauenburg, Andreas Mielcarek, Florian Altegoer, Christopher D. Fage, Uwe Linne, Gert Bange, Mohamed A. Marahiel

**Affiliations:** 1 Department of Chemistry, Biochemistry, Hans-Meerwein Str. 4, Philipps University Marburg, 35043 Marburg, Germany; 2 LOEWE Center for Synthetic Microbiology, Philipps University Marburg, 35043 Marburg, Germany; National Institute of Child Health and Human Development, UNITED STATES

## Abstract

The biosynthesis of iron sulfur (Fe-S) clusters in *Bacillus subtilis* is mediated by a SUF-type gene cluster, consisting of the cysteine desulfurase SufS, the scaffold protein SufU, and the putative chaperone complex SufB/SufC/SufD. Here, we present the high-resolution crystal structure of the SufS homodimer in its product-bound state *(i*.*e*., in complex with pyrodoxal-5ʹ-phosphate, alanine, Cys361-persulfide). By performing hydrogen/deuterium exchange (H/DX) experiments, we characterized the interaction of SufS with SufU and demonstrate that SufU induces an opening of the active site pocket of SufS. Recent data indicate that frataxin could be involved in Fe-S cluster biosynthesis by facilitating iron incorporation. H/DX experiments show that frataxin indeed interacts with the SufS/SufU complex at the active site. Our findings deepen the current understanding of Fe-S cluster biosynthesis, a complex yet essential process, in the model organism *B*. *subtilis*.

## Introduction

Iron sulfur (Fe-S) clusters are amongst the most versatile enzyme cofactors in Nature, as they are involved in cellular respiration, carbohydrate metabolism, DNA repair and various other vital functions throughout all kingdoms of life [1–4]. The biosynthesis of Fe-S clusters must be tightly regulated because of the toxicity of free sulfur and iron. Therefore, the systems for Fe-S cluster biogenesis are mainly conserved from bacteria to human, although elaborate transport systems have diverged through evolution [5–7]. Three distinct systems have been described for prokaryotic cells: *i*.*)* the NIF system (**ni**trogen **f**ixation, [8]), *ii*.*)* the ISC system (**i**ron-**s**ulfur **c**luster, [9]) and *iii*.*)* the SUF system (**su**l**f**ur mobilization, [10]). While the NIF system is specific for nitrogenase maturation in azototrophic bacteria, the ISC and/or SUF systems function in housekeeping in most–if not all–bacteria. In some bacterial species (*e*.*g*., *Escherichia coli*), both systems are found, while others (*e*.*g*., *B*. *subtilis*) rely on a single type of Fe-S machinery [10]. In eukaryotic cells, Fe-S biosynthesis takes place in the mitochondria in an ISC-like system [6] and in plastids in a SUF-like system [11]. In all of these systems, the Fe-S cluster is formed on a scaffold protein before it is transferred to a target apoprotein [12]. A cysteine desulfurase acquires sulfur from cysteine in a pyrodoxal-5ʹ-phosphate (PLP)-dependent reaction and then transfers it as persulfide to the scaffold protein [13,14]. In the *E*. *coli* SUF and eukaryotic systems, auxiliary proteins (SufS/SufE and Nfs1/Isd11, respectively) were found to enhance the activity of the cysteine desulfurase and aid in persulfide transfer [15,16]. The assembly also relies on electron transport, most likely for the reduction of sulfane (S^0^) to sulfide (S^2-^). For this purpose, it was shown that the *E*. *coli* ISC system utilizes ferredoxin [17], while the *E*. *coli* SUF system relies on a scaffold protein (SufB) associated with FAD [15] (in the latter case, however, *in vitro* experiments suggested a role in reduction of ferric iron rather sulfur) [18]. The mechanism of iron insertion remains elusive. It has been suggested that the highly conserved protein frataxin might act as the iron donor, although this role is still under debate. Structural analysis showed that frataxin assumes an α/β fold in which the N-terminal α-helix consists of several acidic residues, commonly referred to as the ‘acidic ridge’ [19–23]. Deletion of frataxin in *Saccharomyces cerevisiae* results in accumulation of iron in the mitochondria and drastically decreases biosynthesis of Fe-S clusters [24]. Frataxin can bind iron and interact with the cysteine desulfurase as well as the Fe-S scaffold protein. These observations led to the suggestion that frataxin could act as an iron chaperone [25]. The latter hypothesis was challenged by the finding that deletion of the *E*. *coli* gene encoding frataxin does not lead to iron accumulation or a decrease in Fe-S clusters as observed in *S*. *cerevisiae*, even though *E*. *coli* frataxin also forms a complex with SufS/SufU homologs IscS/IscU [26,27].

In *Bacillus subtilis* (*Bs*), a frataxin homolog Fra (formerly YdhG) shares only little sequence identity with other frataxins. However, structural analysis of *Bs*Fra showed a conserved α/β sandwich fold with a cluster of acidic residues on the N-terminal α1 and α2 helices, forming an “acidic ridge” [28]. *Bs*Fra was found to bind two equivalents of iron with a moderate *K*_d_ and to interact with the *Thermotoga maritima* (*Tm*) iron-sulfur cluster scaffold protein *Tm*Isu [28]. Based on deletion mutants, it was suggested that *Bs*Fra is a global iron regulator involved in the distribution of iron in *B*. *subtilis* [29]. Previously, the cysteine desulfurase *Bs*SufS and putative scaffold protein *Bs*SufU were characterized *in vivo* and *in vitro*, and it was shown that *BsFra* can be utilized as an iron source [28–31]. We recently demonstrated that *Bs*Fra interacts with the ferrochelatase HemH and is crucial for the incorporation of iron into protoporphyrin [32]. However, the role of frataxin in delivery of iron to SufS/SufU is still poorly understood; in particular, in Fe-S biosynthesis, no interaction between *Bs*Fra and the *Bs*SufS/*Bs*SufU complex has been observed so far. Herein, we demonstrate that *Bs*Fra can indeed interact with *Bs*SufS and *Bs*SufU, as characterized by hydrogen/deuterium exchange (H/DX) experiments. Furthermore, we present the crystal structure of *Bs*SufS and suggest a model for the protein complex consisting of *Bs*SufS/*Bs*SufU/*Bs*Fra.

## Material and Methods

### Protein expression and purification

Plasmids for heterologous expression were previously prepared [30,33]. The heterologous expression of *fra* [29,32], *sufU* [30] and *sufS* [31] was carried out in *E*. *coli* BL21(DE) cells for 20 h at 22°C in LB (lysogeny broth) medium with 50 μg/mL kanamycin and 0.2 mM IPTG. Cells were collected and washed in HEPES buffer A (50 mM HEPES, pH 8, 300 mM NaCl, 5 mM imidazole), treated with DNaseI, and lysed with a French press. The crude extract was cleared by centrifugation (17000 rpm, 4°C, 30 min) and the supernatant was filtered (0.25 μm). The target protein was purified by Ni-affinity chromatography on an FPLC system (NGC Quest, Biorad) using a gradient of 5–100% HEPES buffer B (50 mM HEPES, pH 8, 300 mM NaCl, 250 mM imidazole) over 20 min. The elution fractions were concentrated and further purified by size exclusion chromatography (HiLoad 26/60 Superdex 200, GE Healthcare Life Sciences) in HEPES buffer C (50 mM HEPES, pH 8, 300 mM NaCl). Fractions containing the target protein were concentrated, flash frozen in liquid nitrogen and stored at -80°C in HEPES buffer C supplemented with 10% glycerol.

### Crystallization, data collection, and structure determination

Crystallization was performed by the sitting-drop method at 20°C in 0.6-μl drops consisting of equal parts protein and crystallization solutions. *Bs*SufS crystallized at 20 mg/ml within one week in 0.1 M HEPES, pH 7.5, 50% (v/v) PEG 400. Prior to data collection, crystals were cryoprotected in a solution consisting of the well solution supplemented with 20% glycerol and then flash-frozen in liquid nitrogen. Data were collected under cryogenic conditions at the European Synchrotron Radiation Facility at Beamline ID29. Data were processed with XDS [34] and scaled with CCP4-implemented SCALA [35]. The structure was determined by molecular replacement with PHASER [36], manually built in COOT [37], and refined with PHENIX [38]. The SufS homolog from *Brucella suis* (PDB ID 4W91) was employed as a search model. Figures were prepared with Pymol (www.pymol.org). Coordinates and structure factors were deposited in the Protein Data Bank with the accession code 5J8Q.

### Hydrogen/deuterium exchange experiments

H/DX mass spectrometric analysis of the samples was performed using an automated H/DX setup (Waters) including a two-arm robotic autosampler (LEAP Technologies), an ACQUITY UPLC M-Class System and HDX Manager (Waters). For the exchange reaction, *Bs*Fra, *Bs*SufU, *Bs*SufS, *Bs*Fra/*Bs*SufU, *Bs*Fra/*Bs*SufS, *Bs*Fra/*Bs*SufU/*Bs*SufS, and *Bs*SufU/*Bs*SufS (60 μM final concentration of each component) were individually prepared in H_2_O buffer (25 mM Tris-Cl, pH 7.5, 100 mM NaCl) [32] and pre-cooled to 1°C. For each LC-MS run, 7.5 μL of protein solution was pipetted into a fresh vial on an exchange plate at 25°C and diluted with 61.8 μL of either H_2_O buffer (t0 runs) or D_2_O buffer (exchange runs). After incubation for 15, 30, 60, or 600 s, 55 μL of the reaction solution was transferred to a fresh vial containing 55 μL of quenching solution (400 mM H_3_PO_4_/KH_2_PO_4_, pH 2.2, pre-dispensed and pre-cooled to 1°C for 10 min before the first run). After quenching, 95 μL of the resulting solution was immediately injected into the HDX Manager.

Digestion was done online using an Enzymate BEH Pepsin Column (Waters) at 20°C with water/0.1% formic acid at a flow rate of 100 μL/min. Subsequently peptic peptides were trapped at 0.5°C using a C18 trap column. Separation of peptides was achieved at 0.5°C utilizing a 1 x 100 mm ACQUITY UPLC BEH C18 1.7 μm column (Waters) at a flow rate of 30 μL/min with the following gradient of solvents A (water/0.1% formic acid) and B (acetonitrile/0.1% formic acid): Linear increase from 5–35% B within 7 min, followed by a ramp to 85% B within 1 min and isocratic 85% B for additional 2 min. Finally, the column was washed with 95% B for 1 min and re-equilibrated with 5% B for 5 min.

During separation of peptides using the chromatographic column, the pepsin column was washed by injecting 3 × 80 μL 4% acetonitrile and 0.5 M guanidinium chloride. HDMSe was used for t0 peptide detection and HDMS for exchanged peptides. Lock mass spectra were measured every 45 s using Glu-fibrinopeptide B as a standard ([M+H]^2+^ = 785.8427 m/z). t0 peptide identification was performed using ProteinLynx Global SERVER (Waters) with custom databases and the setting “no enzyme”. Final assignment of deuterium incorporation was done with DynamX 3.0 (Waters).

### Microscale thermophoresis

The determination of binding constants was done by microscale thermophoreis using a Monolith NT.115 instrument (NanoTemper). 100 μM *Bs*Fra was labeled at 8°C overnight using the Monolith NT.115 Protein Labeling Kit RED MALEIMID (cysteine-reactive; NanoTemper). Labeled *Bs*Fra was buffer-exchanged into a binding buffer (25 mM Tris-Cl, 100 mM NaCl, 10 mM 2-mercaptoethanol, pH 7.5) and the concentration was adjusted to 0.25 μM. A serial dilution series of *Bs*SufS, *Bs*SufU, and *Bs*SufS/*Bs*SufU was prepared in the same buffer in a range from 560 μM to 0.030 μM and then mixed 1:1 with labeled *Bs*Fra. The titration was transferred into NT.115 MST Premium Coated capillaries (NanoTemper). Measurements were performed at 25°C, 20% LED power and 20% MST power with a heating time of 30 s and cooling time of 5 s. The binding constant for each interaction was calculated from the average of three measurements using the NT Analysis software (NanoTemper).

### Cysteine Desulfurase Activity Assay

The activity of *Bs*SufS was measured by the amount of sulfide released during conversion of cysteine to alanine. Free sulfide was quantified using *N*,*N*-dimethyl-*p*-phenylenediamine sulfate (DMPD) and FeCl_3_ as described previously [30,31,39]. We incubated 0.5 μM *Bs*SufS with 10 μM *Bs*SufU and 50 μM *Bs*Fra in 25 mM Tris-Cl, pH 7.5, 100 mM NaCl, 5 mM dithiothreitol (DTT) for 5 min at room temperature. The 200 μL reaction was started by addition of l-cysteine (2 mM) and quenched after 10 min by addition of 25 μL of DMPD (20 mM in 7.2 M HCl) and 25 μL FeCl_3_ (30 mM in 1.2 M HCl). After 30 min of incubation in the dark, the absorbance was measured at 670 nm. All reactions were carried out in triplicate.

### Fe-S Biosynthesis Assays

The *Bs*SufS-dependent biosynthesis of Fe-S clusters on *Bs*SufU was assayed in an anaerobic chamber (Coy Laboratories) with forming gas (2% H_2_/98% N_2_) as previously described [30,33]. Briefly, 0.5 μM *Bs*SufS was incubated with 10 μM *Bs*SufU in 25 mM Tris-Cl, pH 7.5, 100 mM NaCl, 5 mM DTT for 5 min at 15°C. Next, 100 μM ammonium iron(II) sulfate was added to the reaction mixture with or without 50 μM *Bs*Fra. The reaction was started by addition of 2 mM cysteine, and Fe-S cluster formation on *Bs*SufU was monitored by UV-Vis at 465 nm (ε_456_ = 5.8 mM^-1^cm^-1^) [40].

## Results

### Crystal structure of *Bs*SufS in the product-bound state

**Fig 1:**
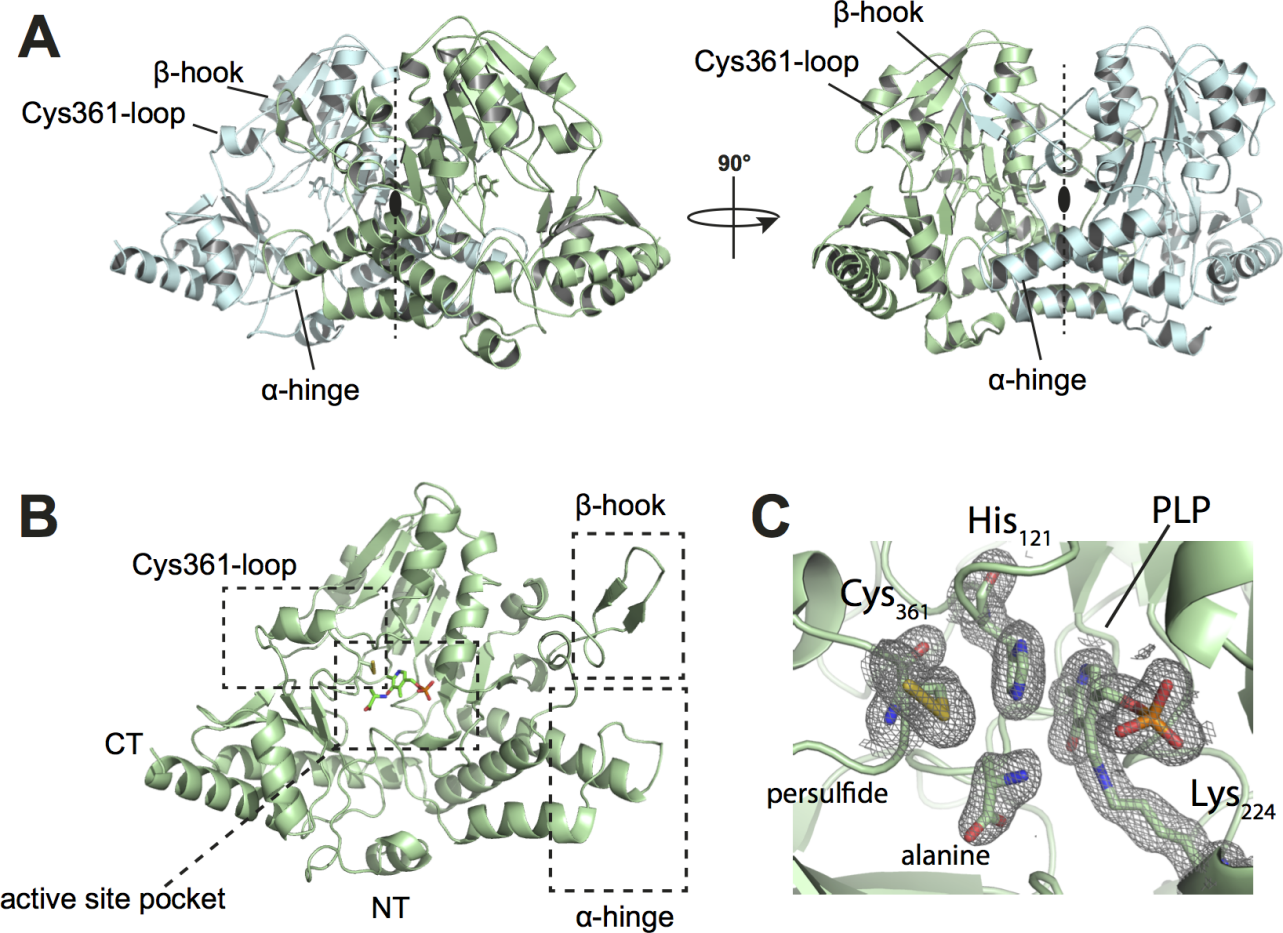
Crystal Structure of the *B*. *subtilis* cysteine desulfurase SufS at 1.7 Å. **(A)** Overall structure of the *Bs*SufS homodimer. The dimer forms a two-fold symmetry axis which covers the active site pocket. One monomer is colored light green and the other, cyan. **(B)** Monomeric *Bs*SufS consists of two domains: The larger, N-terminal domain harbors the PLP binding pocket and the smaller, C-terminal domain contains the flexible Cys361-loop (on which the catalytic Cys361 resides). **(C)** Magnified view of the active site pocket of *Bs*SufS, showing the products alanine and Cys361-persulfide. Pyrodoxal-5ʹ-phosphate is bound to Lys224 as an aldimine. A 2*F*_o_-*F*_c_ map density (grey mesh) is shown, contoured at 1.0 r.m.s.d. N-terminus (NT), C-terminus (CT).

As no structure of *B*. *subtilis* SufS was available at the start of our study, we sought to fill this gap. *Bs*SufS was thus crystallized and its structure was determined to 1.7 Å resolution by molecular replacement using the *B*. *suis* homolog (PDB ID 4W91) [41] as a search model (**Table 1**). One molecule of SufS was found within the asymmetric unit (AU) and could be built to completeness. The crystal structure of *Bs*SufS is highly similar to that of *E*. *coli* SufS (PBD ID 1I29) with a root mean square deviation (r.m.s.d.) of 1.28 Å over 407 Cα atoms (**S1 Fig**). The *Bs*SufS monomer forms a tightly intertwined homodimer with another monomer across the crystallographic symmetry axis (**Fig 1A**). The interface and architecture of the *Bs*SufS homodimer closely resemble those of *E*. *coli* SufS [42].

**Table 1:** Data collection and refinement statistics for *Bs*SufS.

Data collection		
Space group		*P* 31 2 1
Unit cell		
	*a*, *b*, *c* (Å)	93.2, 93.2, 130.5
	α, β, γ (°)	90, 90, 120
Wavelength (Å)		0.9790
Resolution (Å)		46.60–1.70 (1.76–1.70)*
No. of unique reflections		72121 (6911)
Multiplicity		9.7 (9.4)
Completeness (%)		1.00 (0.97)
Mean I/sigma(I)		18.5 (2.7)
*R*_merge_		0.071 (0.753)
**Refinement**		
No. of unique reflections		72119 (6911)
*R*_work_/*R*_free_		0.134/0.159 (0.175/0.212)
No. of atoms		3562
	Protein	3254
	Ligand	15
	Water	293
R.m.s. deviations		
	Angles (°)	1.58
	Bonds (Å)	0.018
Ramachandran allowed (%)		2.9
Ramachandran outliers (%)		0.2
Average *B*-factor (Å^2^)		30.2
	Protein	29.0
	Ligand	26.1
	Water	43.8

*Statistics for the highest-resolution shell are shown in parentheses.

Data were collected from a single crystal.

*Bs*SufS assumes a type I fold of the aminotransferase class V family, consisting of an overall α/β fold that is highly similar to that of *E*. *coli* SufS, IscS, NifS, and CsdA [41,43–46]. The enzyme consists of two domains: A large, N-terminal domain (residues 1–294) and a smaller, C-terminal domain (residues 295–406). In the N-terminal domain, a 7-stranded, parallel β-sheet is sandwiched between several α-helices to form a tightly packed core. This domain harbors the active site pocket, where the cofactor PLP is bound to Lys224 as an aldimine. Furthermore, residues 253–265 (herein referred to as the ‘β-hook’) form a hairpin-like structure that latches on to the other monomer. The small, C-terminal domain consists of a 4-stranded, parallel β-sheet and four α-helices containing the flexible ‘Cys361-loop’ with the nucleophile Cys361 (**Fig 1B**). The dimer interface covers much of the active site pocket and protects PLP and Cys361. We found *Bs*SufS in the product-bound state, with alanine bound near Cys361-persulfide, suggesting that one reaction cycle had occurred.

### *Bs*SufS forms a homodimer in solution

**Fig 2:**
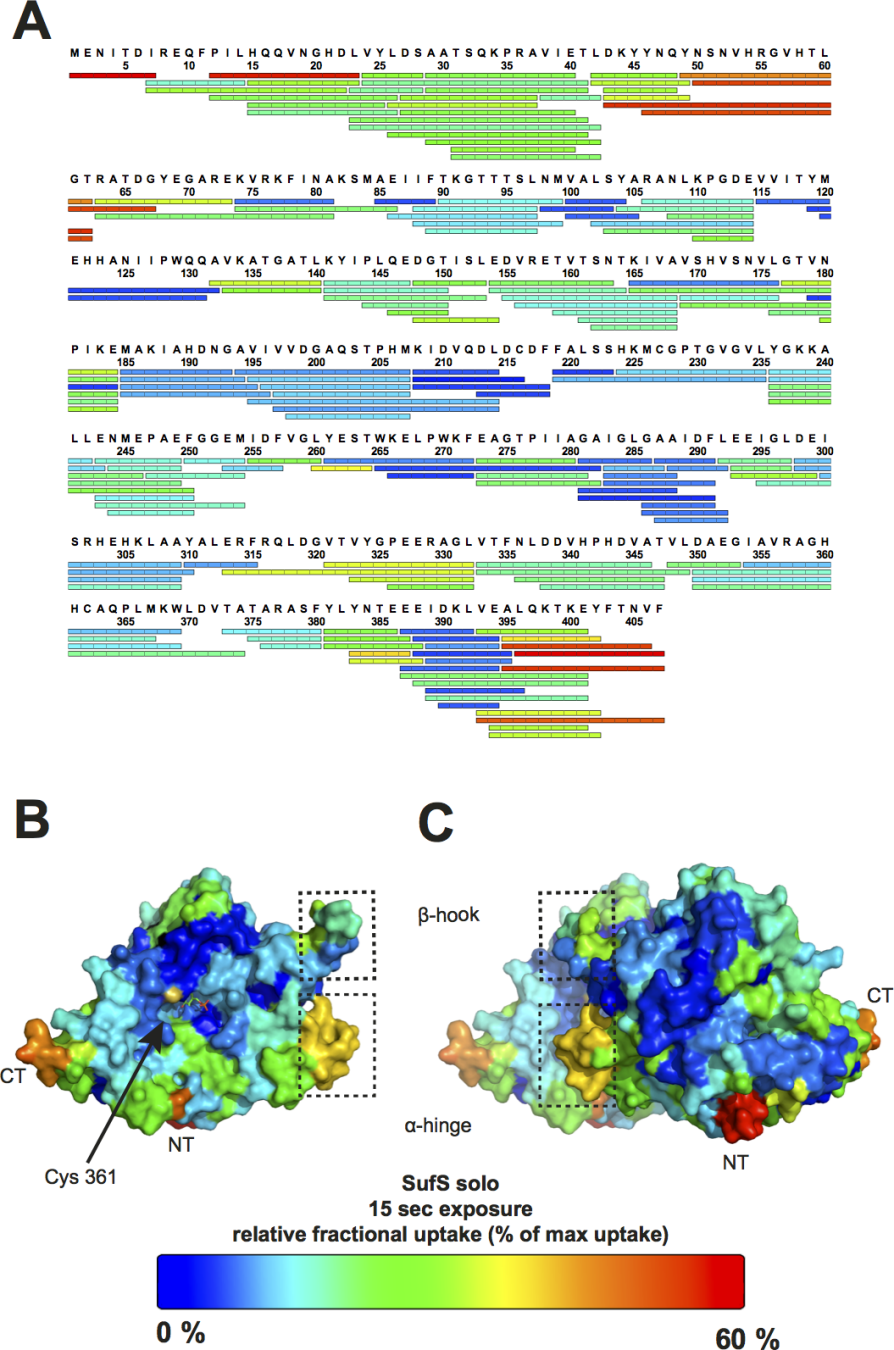
H/DX heat map of *Bs*SufS. The dynamics of *Bs*SufS were analyzed in solution by incubation in deuterated buffer. The relative amount of deuterium incorporated after 15 s, indicated by a color code ranging from blue (low; stable region) to red (high; flexible region), is mapped on the detected peptic peptides of **(A)** *Bs*SufS or mapped onto the surface of the *Bs*SufS **(B)** monomer and **(C)** homodimer. N-terminus (NT), C-terminus (CT).

We performed time-resolved H/DX experiments in order to understand the dynamics of *Bs*SufS in solution. We identified 176 peptides between 5 and 20 amino acids in length, resulting in 98.6% sequence coverage and a redundancy of 4.4 (**Fig 2**). *Bs*SufS was incubated in D_2_O buffer for 15, 30, 60, and 600 s, and the exchange of backbone amide hydrogen to deuterium was measured for each peptide. In good agreement with the expectations from our crystallographic analysis, the solvent-exposed N- and C-termini of *Bs*SufS exchanged readily, whereas most of the core structure was strongly protected (compare to **Fig 1**). Strong protection was observed for *Bs*SufS regions involved in formation of the homodimer interface, validating the existence of the enzyme as a homodimer in solution [47] (**Fig 2B**).

### *Bs*SufU binds to the *Bs*SufS homodimer

**Fig 3:**
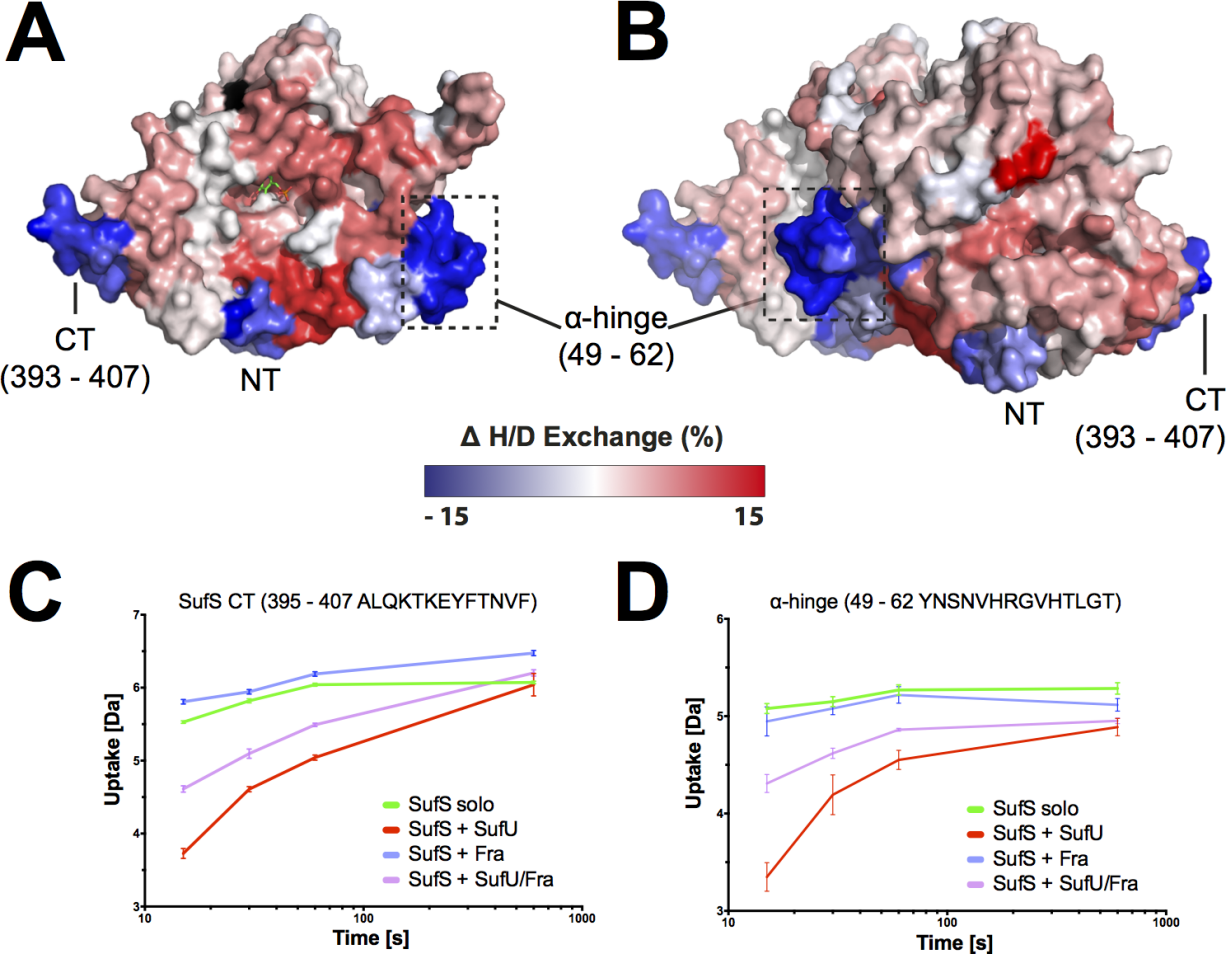
*Bs*SufU alters the H/D exchange of *Bs*SufS upon binding. Changes in relative fractional deuterium uptake of *Bs*SufS after incubation with *Bs*SufU for 15 s in D_2_O buffer compared to *Bs*SufS alone were mapped onto the surface of the *Bs*SufS **(A)** monomer and **(B)** homodimer. A decrease (blue) in deuterium uptake signals protection (*i*.*e*., a binding event), whereas an increase (red) signals a structural rearrangement. Black regions were not detected. Binding of *Bs*SufU to **(C)** the C-terminus and **(D)** the α-hinge of *Bs*SufS as a function of deuterium uptake over time. Color code: *Bs*SufS alone (green), *Bs*SufS + *Bs*SufU (red), *Bs*SufS + *Bs*Fra (blue), and BsSufS + *Bs*SufU/*Bs*Fra (violet). N-terminus (NT) and C-terminus (CT).

Next, we sought to investigate the interaction interface and conformational dynamics of SufS and SufU. Therefore, SufS was incubated with SufU in deuterated buffer. Upon completion of the H/DX reaction and peptic digest, we observed that several peptides of SufS showed a change in deuterium uptake when compared to their counterparts from H/DX with SufS alone (see above). Most notably, the C-terminal α-helix of SufS showed a high degree of protection in the presence of SufU (**Fig 3**). This observation agrees with recent findings that the C-terminus of *E*. *coli* IscS is required for interaction with IscU [48,49]. In addition, residues 50–63 of SufS (herein referred to as the ‘α-hinge’) were protected against deuterium incorporation, suggesting the presence of a second SufU interaction site at the SufS homodimer interface.

**Fig 4:**
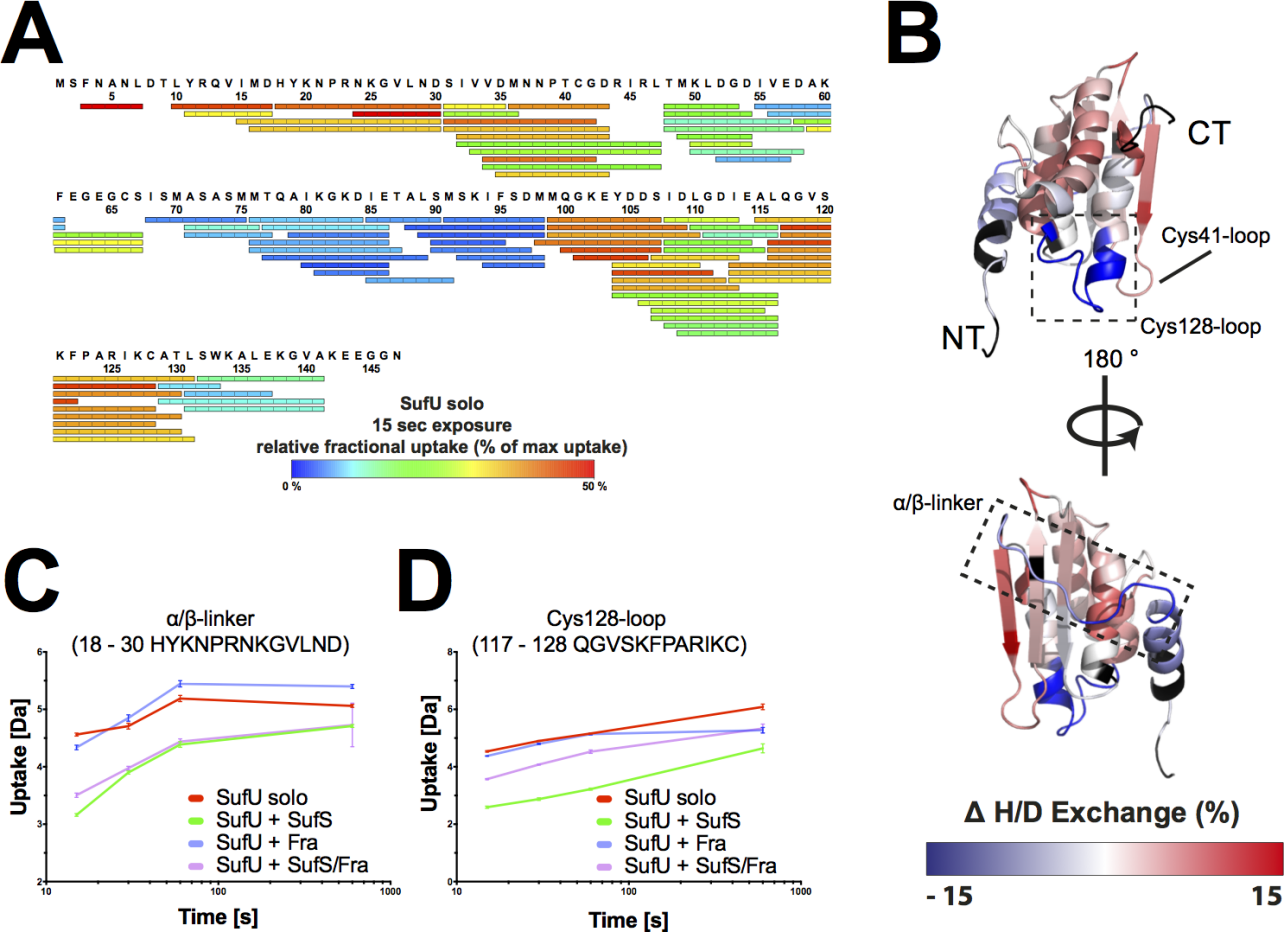
*Bs*SufS alters the H/D exchange of *Bs*SufU upon binding. **(A)** Detected peptic peptides of *Bs*SufU with the relative fractional uptake after 15 s of incubation in deuterated buffer. **(B)** Changes in the relative fractional deuterium uptake of *Bs*SufU after incubation with *Bs*SufS for 15 s in D_2_O buffer compared to *Bs*SufU alone were mapped onto the surface of *Bs*SufU (PDB ID 2AZH). The heat map represents the differences in deuterium uptake compared to *Bs*SufU alone. A decrease (blue) in deuterium uptake signals protection (*i*.*e*., a binding event), whereas an increase (red) signals a structural rearrangement. Black regions were not detected. Binding of *Bs*SufU to **(C)** the α/β-linker and **(D)** the Cys128-loop of *Bs*SufS as a function of deuterium uptake over time. Color code: *Bs*SufU alone (red), *Bs*SufU + *Bs*SufS (green), *Bs*SufU + *Bs*Fra (blue), and BsSufU + *Bs*SufS/*Bs*Fra (violet). N-terminus (NT) and C-terminus (CT).

We then utilized our H/DX approach to analyze changes in SufU upon binding SufS. We identified 87 peptides, which covered 93% of the sequence with a redundancy of 6.0 (**Fig 4**). The NMR solution structure of apo-SufU (PDB ID 2AZH) reveals a compact, α/β fold. Our H/DX analysis supports this structure in solution, as medium to low H/D exchange was observed for the core of SufU, whereas high H/D exchange was observed for an extended loop region between the N-terminal helix α1 and strand β1 (the ‘α/β linker’) as expected for a solvent-exposed, unstructured region. In the presence of SufS, the ‘α/β-linker’ of SufU is strongly protected from deuterium uptake, which indicates that SufS interacts with SufU at this location (**Fig 4C**). Residues 117–130, which form a loop carrying Cys128 (the ‘Cys128-loop’), are also protected from deuterium uptake, suggesting a second SufS binding site. Therefore, we conclude that the ‘α/β-linker’ and ‘Cys128-loop’ of SufU bind to the C-terminus and ‘α-hinge’ of SufS, respectively, bringing the active site of SufS and SufU into close proximity. A similar scenario has been proposed for the interaction of IscU and IscS in *E*. *coli* [49].

### Binding of *Bs*SufU to *Bs*SufS induces conformational changes in both proteins

Next, we analyzed the H/DX data for changes in the dynamic behavior of SufS in the presence of SufU. Significantly higher H/D exchange rates were observed for the active site pocket of SufS when SufU was present, particularly at the ‘Cys361-loop’ and ‘β-hook’ of SufS (**Fig 3**). These observations strongly suggest that the SufS homodimer opens in the presence of SufU, allowing the ‘Cys361-loop’ to move freely. This is in agreement with findings in *E*. *coli*, where the ‘Cys361-loop’ of IscS undergoes a major, 14-Å movement during transfer of sulfur to IscU [48,49].

Our data also show that, in the presence of SufS, SufU undergoes significant structural rearrangements at the ‘α-helical bundle’ (*i*.*e*., helices α2, α3, and α5) and ‘β-sheet surface’ (*i*.*e*., residues 32–47). The active site of SufU contains four residues (*i*.*e*., Cys41, Asp43, Cys66, and Cys128) that coordinate a structurally important zinc ion [30,50]. In particular, the ‘Cys41-loop’ showed a significantly increased deuterium uptake. To summarize, the interaction of *Bs*SufS with *Bs*SufU induces structural rearrangements near the active sites of both proteins, potentially facilitating persulfide transfer. This notion supports the 40-fold increase in *Bs*SufS desulfurase activity previously observed upon interaction with *Bs*SufU [30,31,47,50].

### *Bs*Fra binds to *Bs*SufU and *Bs*SufS

**Fig 5:**
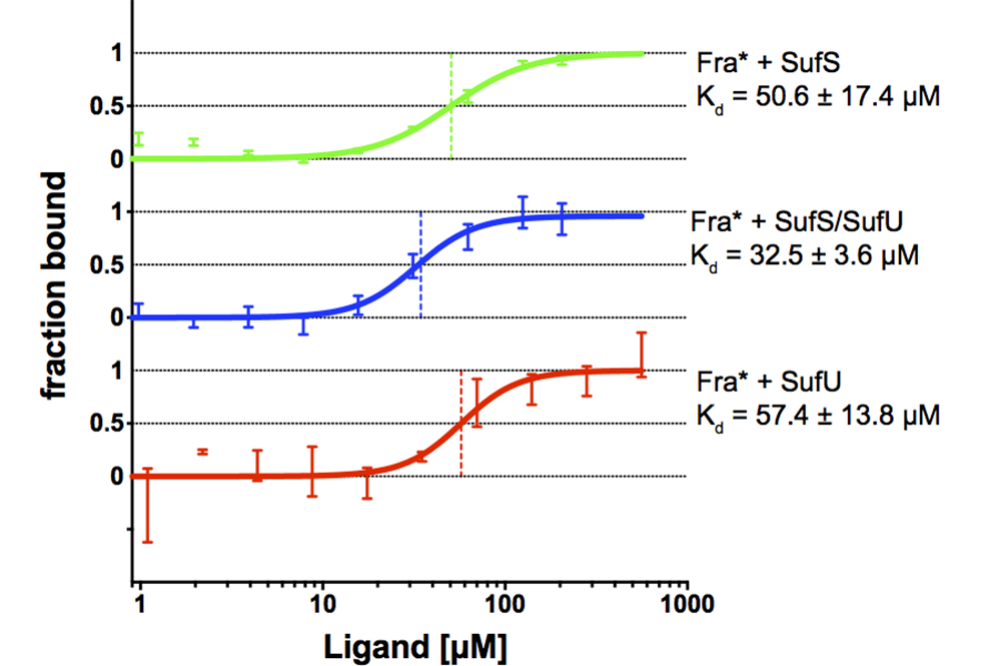
Characterization of the affinity of *Bs*Fra for *Bs*SufS, *Bs*SufU, and *Bs*SufS/*Bs*SufU using microscale thermophoresis. MST binding curve from the interaction of fluorophore-labeled *Bs*Fra with **(A)** *Bs*SufS, **(B)** *Bs*SufS/*Bs*SufU, and **(C)** *Bs*SufU. A Hill model was applied for *K*_d_ determination. Fra* indicates fluorophore-tagged frataxin.

After demonstrating the dynamic behavior and interaction of the *Bs*SufS/*Bs*SufU complex, we sought to integrate *B*. *subtilis* frataxin into the picture. We conducted microscale thermophoresis (MST) experiments between fluorophore-labelled *Bs*Fra and *Bs*SufU, *Bs*SufS, and *Bs*SufS/*Bs*SufU, and found that while *Bs*Fra binds its partners fairly weakly: *Bs*SufU (*K*_d_ = 57.4 ± 13.8 μM), *Bs*SufS (*K*_d_ = 50.6 ± 17.4 μM) and the *Bs*SufS/*Bs*SufU complex (*K*_d_ = 32.5 ± 3.6 μM) (**Fig 5**). In contrast, the interaction of *Bs*SufS with *Bs*SufU (*K*_d_ = 2.63 μM) [31] and *Bs*Fra with *Bs*HemH (*K*_d_ = 1.63 μM) [32] were found to be significantly tighter. Nevertheless, our affinity measurement for *Bs*Fra with *Bs*SufS/*Bs*SufU is of the same order of magnitude as that for the homologous *E*. *coli* system comprised of CyaY and IscS (*K*_d_ = 18.5 μM) [51].

**Fig 6:**
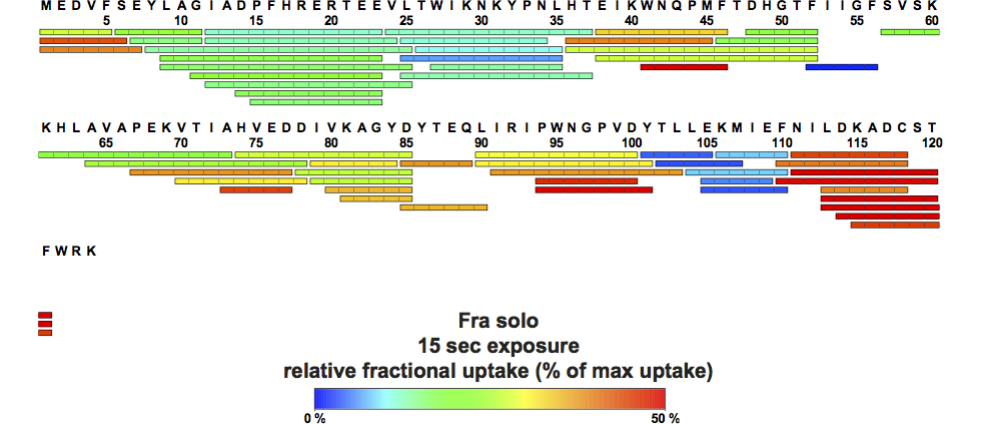
Peptic peptides of *Bs*Fra detected from H/DX measurements. The relative amount of deuterium incorporated after 15 s, indicated by a color code ranging from blue (low; stable region) to red (high; flexible region), is mapped on the detected peptic peptides of *Bs*Fra.

To further characterize the interaction of *Bs*Fra with its partners we applied H/DX experiments. Analysis of *Bs*Fra alone yielded 61 unique peptides with 98% sequence coverage and a redundancy of 4.7 (**Fig 6**). Human, *S*. *cerevisiae*, and *E*. *coli* frataxin (PDB ID 3S4M, 2GA5, and 2EFF, respectively) assume an α/β fold in which two α-helices are stacked against a 6-stranded β-sheet, with the N-terminal α-helix harboring several acidic residues (the ‘acidic ridge’) [21]. *Bs*Fra (PDB ID 2OC6) has an additional short, N-terminal helix (herein referred to as ‘α1-helix’) followed by the helix carrying the ‘acidic ridge’ (compare **S2 Fig**). The ‘α1-helix’ shows medium to high deuterium uptake in *Bs*Fra, suggesting that in solution it can move freely. Furthermore, a helix connecting β4 to β5 (the ‘EDDI-helix’) is present in *Bs*Fra but not homologous frataxin structures [19–21]. A high rate of H/D exchange was observed for this ‘EDDI-helix’, especially for residues E75, D76, D77, and I78.

**Fig 7:**
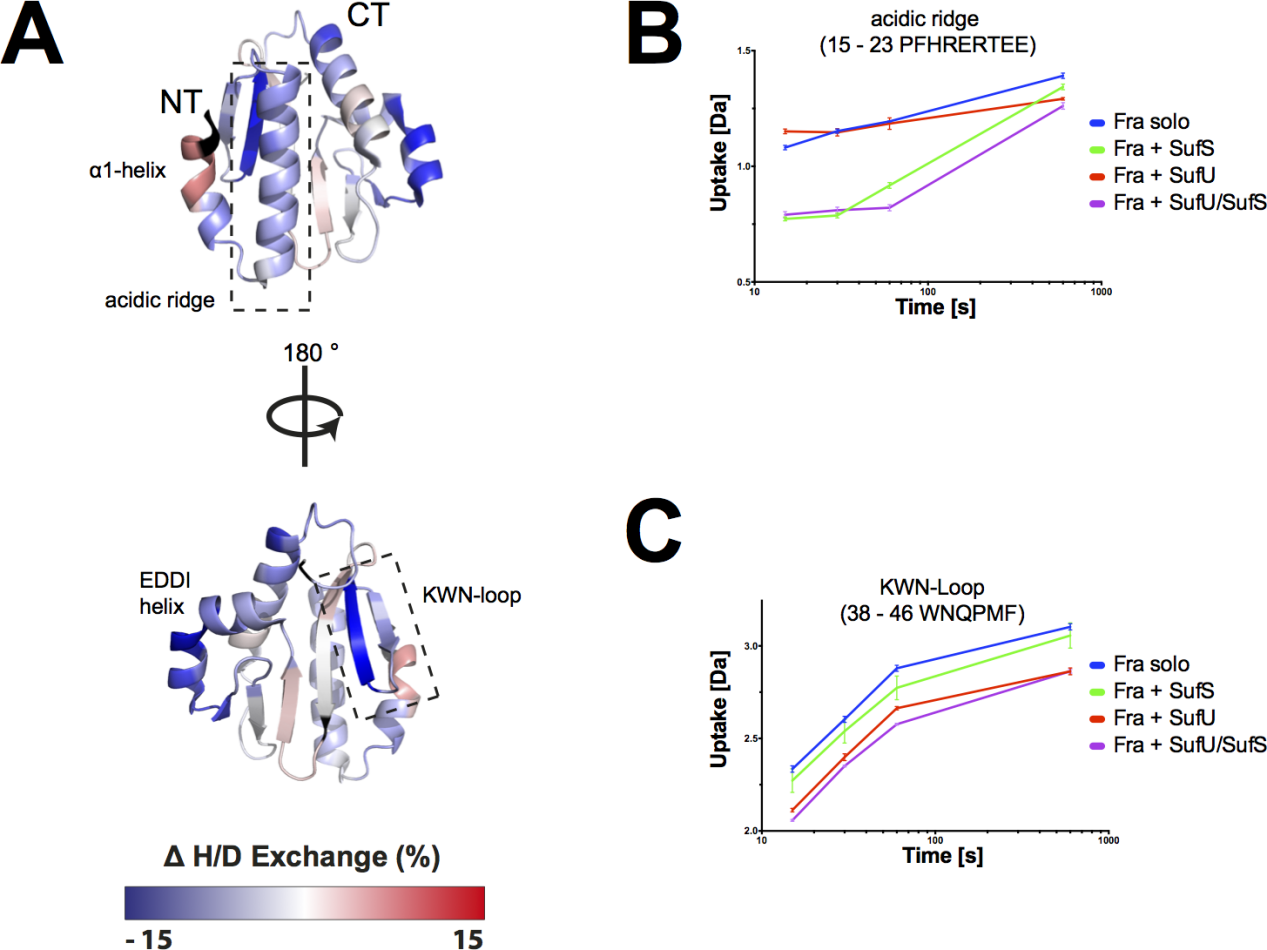
H/DX analysis of *Bs*Fra upon binding *Bs*SufU/*Bs*SufS. **(A)** Changes in the relative fractional deuterium uptake of *Bs*Fra after incubation with *Bs*SufS/*Bs*SufU for 15 s in D_2_O buffer compared to *Bs*Fra alone were mapped onto the surface of *Bs*Fra (PDB ID 2OC6). The heat map represents the differences in deuterium uptake compared to *Bs*Fra alone. A decrease (blue) in deuterium uptake signals protection (*i*.*e*., a binding event), whereas an increase (red) signals a structural rearrangement. Black regions were not detected. Binding of *Bs*SufS/BsSufS to **(B)** the ‘acidic ridge’ and **(C)** the KWN-loop of *Bs*Fra as a function of deuterium uptake over time. Color code: *Bs*Fra alone (blue), *Bs*Fra + *Bs*SufS (green), *Bs*Fra + *Bs*SufU (red), and BsFra + *Bs*SufU/*Bs*SufS (violet). N-terminus (NT) and C-terminus (CT).

We repeated the H/DX experiments of *Bs*Fra in the presence of *Bs*SufU (**S3 Fig**), *Bs*SufS (**S4 Fig**), and the *Bs*SufS/*Bs*SufU complex (**Fig 7**). These measurements were compared with those of *Bs*Fra alone for differences in deuterium uptake. In the presence of *Bs*SufU, we observed a decrease of H/D exchange in β-strands 1 and 2 of *Bs*Fra (residues 37–46, herein referred to as the ‘KWN-loop’), which harbor residues K40, W41, and N42 **(Fig 7C)**. As we found that the same residues were protected in the reaction of *Bs*Fra and *Bs*SufU, we conclude that *Bs*Fra binds to *Bs*SufU at the ‘KWN-loop’ area. The involvement of the ‘KWN-loop’ in the interaction with *Bs*SufU is in agreement with previous studies in which mutations of in this region disrupted the interaction [52–55]. In the presence of both *Bs*SufU and *Bs*SufS, *Bs*Fra was additionally to the KWN loop protected from H/D uptake at the ‘acidic ridge’ (residues 9–25) (**Fig 7**and **S4 Fig**), suggesting an interaction with *Bs*SufS. This is in accordance with previously published work on *E*. *coli* IscS/CyaY [51,56]. Furthermore, *Bs*SufS induces an increased rate of deuterium uptake at the N-terminal ‘α1-helix’ of *Bs*Fra (**Fig 7**). We propose that, upon binding of *Bs*Fra to *Bs*SufS, the ‘α1-helix’ undergoes a structural rearrangement. Interestingly, *Bs*Fra was protected from deuterium uptake at the C-terminus and ‘EDDI helix’ (residues 74–85) compared to the protein alone. However, this was found in the *Bs*Fra/*Bs*SufS and *Bs*Fra/*Bs*SufU reactions (**Fig 7**, **S3 Fig** and **S4 Fig**), suggesting that the either protein can stabilize these highly dynamic areas (compare **Fig 6**). Taken together, the results indicate that *Bs*Fra binds to *Bs*SufS and *Bs*SufU as well as to the *Bs*SufS/*Bs*SufU complex. A similar binding of frataxin to a cysteine desulfurase/scaffold protein complex was described in *E*. *coli* [51,56].

### Binding of *Bs*Fra to *Bs*SufU/*Bs*SufS induces conformational changes in all three proteins

**Fig 8:**
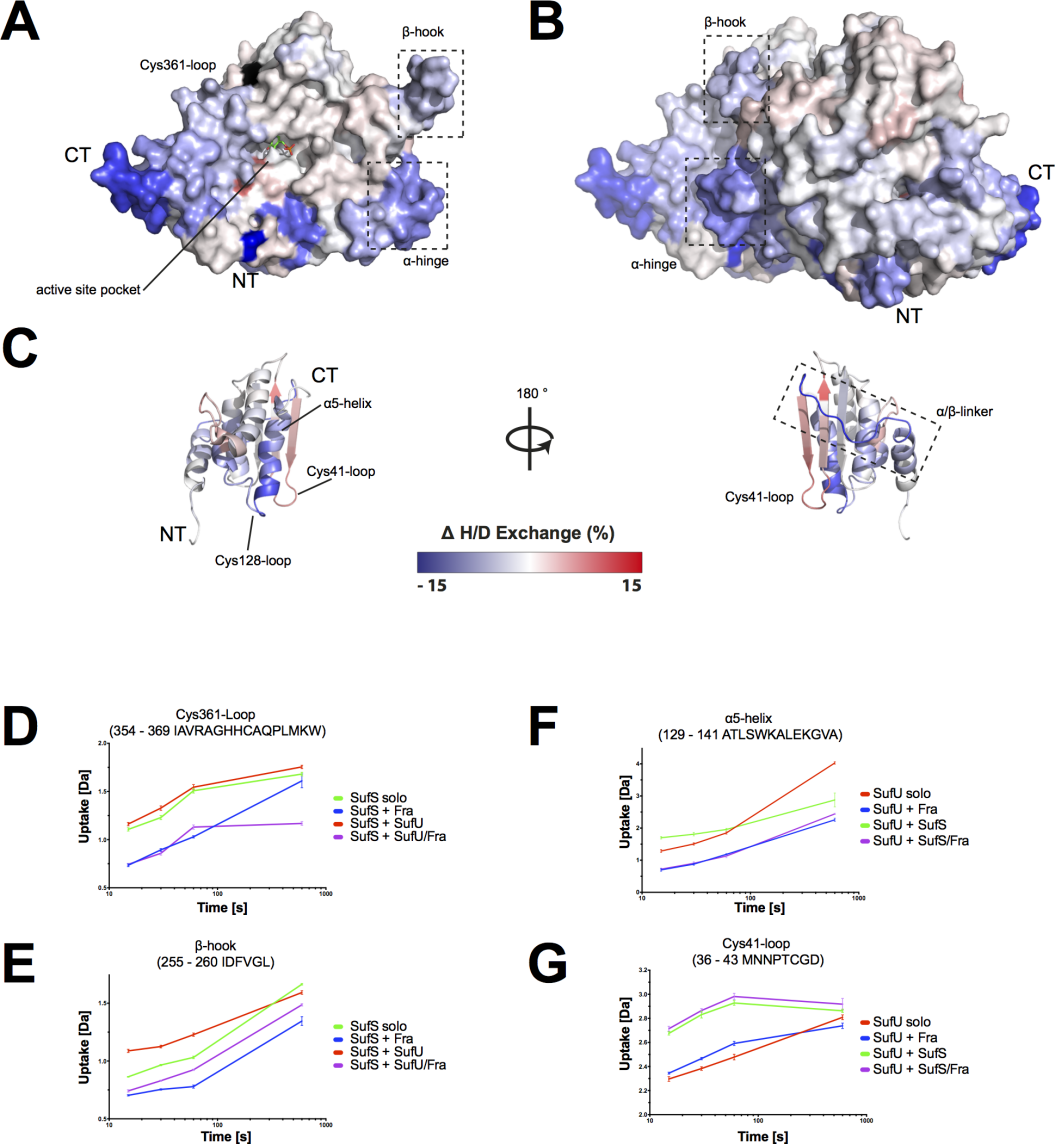
*Bs*Fra alters the H/D exchange of *Bs*SufS and *Bs*SufU upon binding the *Bs*SufS/*Bs*SufU complex. Changes in the relative fractional deuterium uptake of *Bs*SufS/*Bs*SufU after incubation with *Bs*Fra for 15 s in D_2_O buffer compared to *Bs*SufS and *Bs*SufU alone were mapped onto the surface of the *Bs*SufS **(A)** monomer and **(B)** homodimer as well as **(C)** *Bs*SufU (PBD ID 2AZH). The heat map represents the differences in deuterium uptake compared to the solo incubation. A decrease (blue) in the uptake signals protection (*i*.*e*., a binding event), whereas an increase (red) signals a structural rearrangement. Black regions were not detected. Changes in **(D)** the SufS Cys361-loop and **(E)** the β-hook as a function of deuterium uptake over time. Color code: *Bs*SufS alone (green), *Bs*SufS + *Bs*Fra (blue), *Bs*SufS + *Bs*SufU (red), and BsSufS + *Bs*SufU/*Bs*Fra (violet). Changes in **(F)** the SufU α5-helix and **(G)** the Cys41-loop as a function of deuterium uptake over time. Color code: *Bs*SufU alone (red), *Bs*SufU + *Bs*Fra (blue), *Bs*SufU + *Bs*SufS (green), and BsSufU + *Bs*SufS/*Bs*Fra (violet). N-terminus (NT) and C-terminus (CT).

Next, we tested whether the binding of *Bs*Fra would alter the interaction of *Bs*SufS with *Bs*SufU. We analyzed the H/DX data of the *Bs*Fra/*Bs*SufU/*Bs*SufS interaction and compared it to that of the *Bs*SufS/*Bs*SufU complex and proteins alone (see above). The scaffold protein *Bs*SufU showed decreased deuterium uptake at the C-terminal helix (‘α5-helix’), indicating binding to *Bs*Fra (**Fig 8 and S3 Fig**). The cysteine desulfurase *Bs*SufS showed protection from H/D exchange at the ‘Cys361-loop’ and the ‘β-hook’ when *Bs*Fra was present (**Fig 8**), also indicating a binding event. Several positively charged residues are located in this region (*e*.*g*., R356, H359, H360, and K367) and may bind the negatively charged residues of the ‘acidic ridge’ of *Bs*Fra (see above). In contrast to the reaction of *Bs*SufS/*Bs*SufU with *Bs*Fra, no increase in deuterium uptake was observed at the active site pocket of *Bs*SufS when *Bs*Fra was present (compare **Fig 8**to **Fig 3**). The *Bs*SufU binding site on *Bs*SufS (see above) was still observable, supporting the idea of simultaneous binding.

We propose that *Bs*Fra binds via the ‘acidic ridge’ to the positively charged ‘Cys361-loop’ and ‘β-hook’ of *Bs*SufS. *Bs*Fra may act as a clamp at the *Bs*SufS homodimer interface, locking the ‘Cys361-loop’ and ‘β-hook’ and thus preventing the opening of the *Bs*SufS active site pocket. Furthermore, *Bs*Fra binds via its ‘KWN-loop’ to the ‘β-sheet surface’ of *Bs*SufU. A similar binding site was suggested for *E*. *coli* IscS/CyaY [51,56].

**Fig 9:**
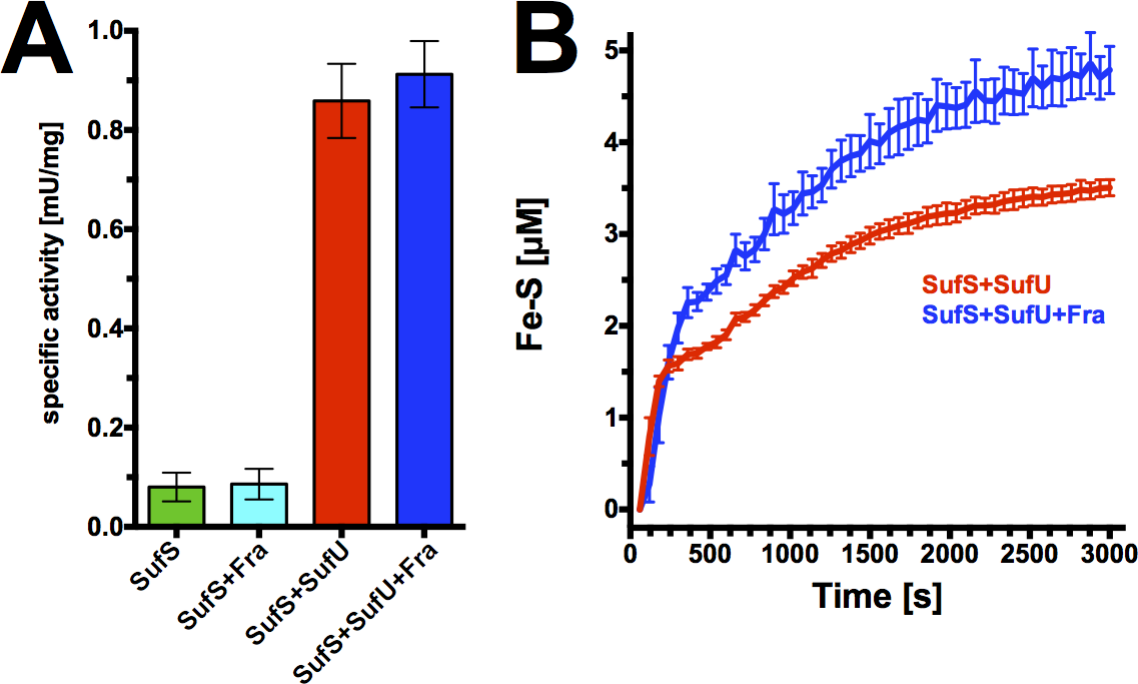
Sulfur transfer and Fe-S cluster biosynthesis assays of *Bs*SufS and *Bs*SufU in the presence of *Bs*Fra. **(A)** The specific activity of *Bs*SufS was measured *in vitro* by the release of sulfide from cysteine. The activity of *Bs*SufS (green) is unaffected by *Bs*Fra (cyan), but is greatly increased by the presence of *Bs*SufU (red). The addition of *Bs*Fra to *Bs*SufS/*Bs*SufU does not affect the activity of *Bs*SufS. **(B)** *In vitro* biogenesis of Fe-S clusters by *Bs*SufS/*Bs*SufU over time (red). Addition of *Bs*Fra (blue) results in a very slight increase in cluster yield.

To test whether the binding of *Bs*Fra to the *Bs*SufS/*Bs*SufU complex alters the activity of *Bs*SufS we conducted cysteine desulfurase activity assays *in vitro*. We found that *Bs*SufU greatly enhances the activity of *Bs*SufS, in agreement with previous reports [30,33]. The introduction of *Bs*Fra, however, does not appear to affect the activation of *Bs*SufS by *Bs*SufU (**Fig 9**). This is in agreement with the *E*. *coli* IscS/CyaY interaction, where conversion of cysteine to alanine by IscS is not affected by CyaY [56]. We next analyzed whether frataxin has an effect on the formation of Fe-S clusters *in vitro*. In an assay described by Albrecht and colleagues [30], we incubated *Bs*SufS and *Bs*SufU in the presence of ferrous iron and cysteine and monitored the formation of Fe-S clusters by UV-Vis spectroscopy. When *Bs*Fra was present in the reaction mixture the initial rate of formation was unaffected, although we observed a very minor increase in the yield of Fe-S clusters formed on *Bs*SufU. Such a subtle change could indicate that either *Bs*Fra does not efficiently donate iron to *Bs*SufU under assay conditions or that its presence is redundant under the supplied concentration of iron. Additionally, other, unidentified components may be required for efficient transfer.

## Discussion

**Fig 10:**
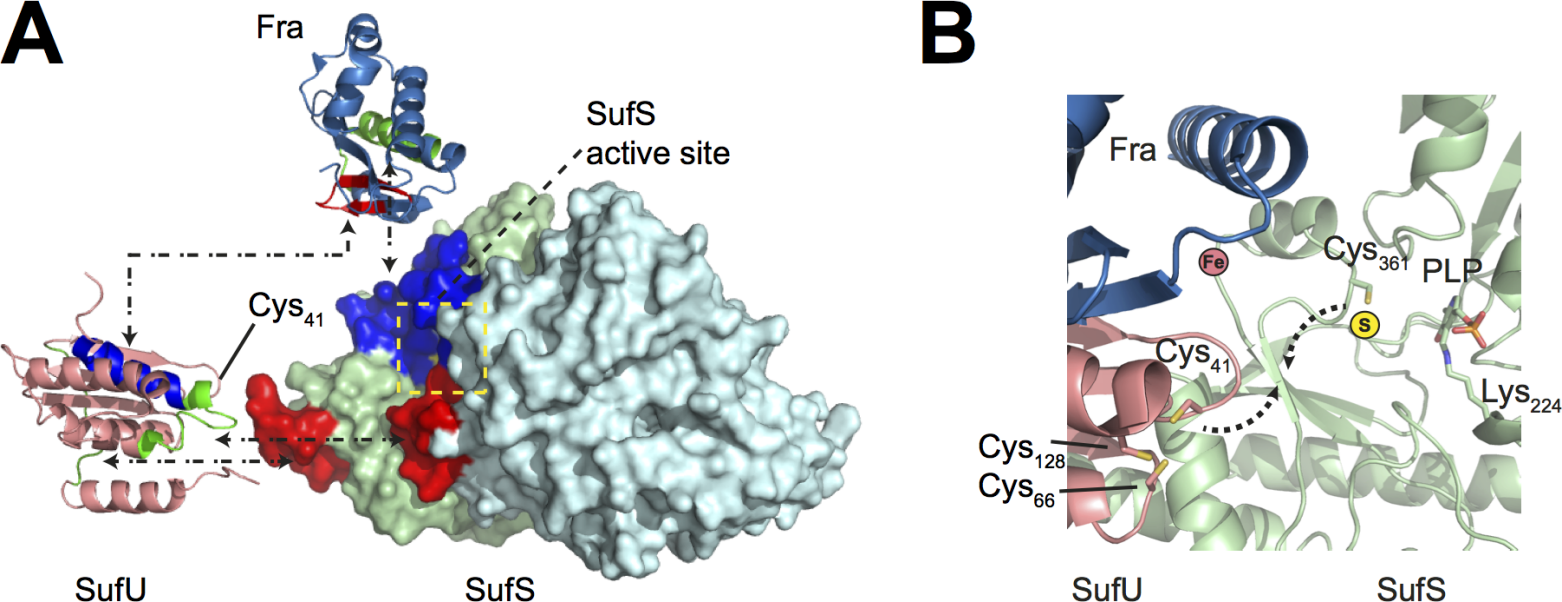
Proposed model for the interaction of *Bs*Fra/*Bs*SufU/*Bs*SufS. **(A)** Based on the H/DX results, we identified the binding sites of *Bs*SufU (PDB ID 2AZH, shown in salmon), *Bs*Fra (PDB ID 2OC6, shown in sky blue) and the *Bs*SufS homodimer (light green and cyan) in the *Bs*Fra/*Bs*SufS/*Bs*SufU complex. The binding epitopes are highlighted according to the color of the respective interaction partner (*i*.*e*., residues protected by *Bs*Fra are shown in blue, those by *Bs*SufS in green, and those by *Bs*SufU in red). **(B)** Proposed binding of *Bs*Fra and *Bs*SufU to *Bs*SufS. The binding of *Bs*SufU to *Bs*SufS brings the SufS residue Cys361 and the SufU residue Cys41 into close proximity for persulfide transfer. While binding of *Bs*Fra to *Bs*SufS does not affect the transfer of persulfide from *Bs*SufS to *Bs*SufU, it brings the putative iron binding site into close proximity to the SufS/SufU active site.

*Bacillus subtilis* has five paralogous cysteine desulfurase genes, namely *nifS* [57], *yrvO* [58], *nifZ* [59] and *ycbU* [60]. However, only the product of the *sufS* gene is involved in biosynthesis of Fe-S clusters. Until now, no structural information was available on the *B*. *subtilis* cysteine desulfurase. We were able to determine the crystal structure of the *Bs*SufS dimer to 1.7 Å resolution, revealing high structural homology to its *E*. *coli* counterpart. To better understand the conformational dynamics of SufS and its interaction with SufU and Fra, we conducted H/DX experiments with proteins from the Gram-positive model organism *B*. *subtilis*. Many insights into the SUF system were previously obtained from studies on *E*. *coli*, in which the SUF system only acts as a backup under stress conditions [7]. The ISC system mediates the major “housekeeping” functions for Fe-S cluster biosynthesis in *E*. *coli* [9]. In *B*. *subtilis*, we find similarities to both *E*. *coli* ISC and SUF systems [30,31,33]. We applied hydrogen/deuterium exchange experiments on *Bs*SufS in order to investigate structural dynamics upon its interaction with the putative iron delivery protein *Bs*Fra and the scaffold protein *Bs*SufU. H/DX detects the exchange of hydrogen on the backbone amides with deuterium in the solvent, where the exchange rate of highly dynamic or surface-exposed areas is rapid compared to residues that are buried in the protein core or otherwise protected. Investigation of *Bs*SufS confirmed the homodimer interface in solution and suggested that the enzyme is tightly packed. Nevertheless, the strength of H/DX lies in its ability to detect changes in residues following a binding event. We showed that the C-terminal region and ‘α-hinge’ of *Bs*SufS interact with the long ‘α/β-linker’ and ‘Cys128-loop’ of *Bs*SufU (**Fig 10**). This interaction is accompanied by an opening of the *Bs*SufS dimer interface and rearrangement of the ‘Cys361-loop’. *Bs*SufU, on the other hand, showed H/D exchange in the loop carrying Cys41 and Asp43. *Bs*SufU fits on the *Bs*SufS interaction site, which brings the ‘Cys361-loop’ of *Bs*SufS and the ‘Cys41-loop’ of *Bs*SufU into close proximity for persulfide transfer.

The *E*. *coli* SufS/SufE interaction was previously characterized by H/DX [61] and the crystal structure of the homologous *E*. *coli* CsdA/CsdE enzyme complex was solved recently [41]. In both cases, SufE/CsdE binds near the active site of SufS/CsdA to accept the sulfur as persulfide, which is then passed on to the scaffold protein complex SufBC_2_D of the *E*. *coli* SUF system. *E*. *coli* SufB has been shown to act as a scaffold and carries an FAD binding site that is possibly important for the reduction of Fe^3+^ to Fe^2+^ in the generation of Fe-S clusters [18]. In *B*. *subtilis*, the roles of SufB, SufC, and SufD are still unclear. *Bs*SufB, though conserved, lacks the Fe-S cluster and FAD binding sites, suggesting an important role which differs from that of its *E*. *coli* homolog. We hypothesize that the *Bs*SufBCD enzyme complex is involved in later steps of Fe-S cluster maturation, similar to the *E*. *coli* HscA/HscB proteins of the ISC system [62–64]. Taken together, our data suggest that binding of *Bs*SufU to *Bs*SufS activates the latter for transfer of sulfur from persulfide to the active site of *Bs*SufU.

In the present study, we further established an interaction between *Bs*SufS/*Bs*SufU and the *B*. *subtilis* frataxin homolog *Bs*Fra. Previous biochemical characterization showed that *Bs*Fra can bind ferric and ferrous iron, and plays a role in Fe-S cluster biosynthesis and transfer to a target protein [28,29]. The frataxin family has been extensively studied in *E*. *coli*, *S*. *cerevisiae*, and *H*. *sapiens*. Generally, this family consists of a conserved α/β sandwich fold and harbors several acidic residues at the N-terminal α-helix and the first β-sheet (**S2 Fig**). *In vitro* enzyme assays measuring the rate at which Fe-S clusters form on the scaffold protein in the presence of a cysteine desulfurase and frataxin highlights a discrepancy: While frataxin inhibits cluster formation in the *E*. *coli* system [51,56], it enhances the rate of cluster formation in the yeast system [65]. It was shown that the cysteine desulfurase determines whether frataxin acts as an inhibitor or activator [66]. It was further shown that a single point mutation on the scaffold protein renders *E*. *coli* as frataxin-dependent and *S*. *cerevisiae* as frataxin-independent [67–69]. We conducted analogous assays with *Bs*Fra and found that, in contrast to *E*. *coli* and *S*. *cerevisiae* homologs, it did not appear to alter the initial rate of cluster formation, but resulted in a very minor increase in cluster yield. The cluster formed on *Bs*SufU is highly labile and most likely degrades to an uncharacterized species upon isolation of holo-*Bs*SufU [30,50]. That we were unable to isolate holo-*Bs*SufU may suggest that our current model for Fe-S biosynthesis includes only the minimal number of participants.

We performed H/DX experiments to analyze the interaction of *Bs*SufS and *Bs*SufU with *Bs*Fra. We observed binding of *Bs*Fra to *Bs*SufU and *Bs*SufS, which did not change in the presence of *Bs*SufU/*Bs*SufS and therefore indicates formation of a *Bs*Fra/*Bs*SufU/*Bs*SufS complex in solution. The binding epitope of *Bs*Fra associates with the ‘Cys361-loop’ and ‘β-hook’ motifs of *Bs*SufS **(Fig 10)**. The interaction points between *Bs*Fra and *Bs*SufU are the ‘KWN-loop’ and ‘β-sheet surface’, respectively. The short ‘α1-helix’ of *Bs*Fra appears to rearrange upon binding *Bs*SufS, forming an extended α1-α2 helix hybrid, which then fits in the groove of *Bs*SufS/*Bs*SufU. This interaction brings the putative iron binding site of *Bs*Fra into close proximity to the active site of *Bs*SufU, and it is reasonable to propose that iron incorporation is mediated by the ‘acidic ridge’ of *Bs*Fra. Previous SAXS measurements of *E*. *coli* IscS/IscU/CyaY revealed a similar binding mode [51], and characterization of the interaction of frataxin with the ferrochelatase established that *Bs*Fra binds with its acidic residues in a similar fashion to the iron acceptor *Bs*HemH [32]. In *B*. *subtilis*, it is well established that frataxin is involved in incorporating iron into the nascent Fe-S cluster *in vivo* [33], but whether frataxin also serves as an intracellular iron carrier remains elusive.

We determined that *Bs*SufU binds *Bs*SufS and further showed how *Bs*Fra binds to the *Bs*SufU/*Bs*SufS complex as well as the individual proteins. In accordance with what is known about *E*. *coli* IscS/IscU/CyaY [51,56], the interaction of *Bs*SufU/*Bs*SufS is tight compared to that of *Bs*Fra with the complex. We assume that the interaction of *Bs*Fra to the complex is transient and that *Bs*Fra competes with additional participants of the Fe-S biogenesis pathway, as is known for *E*. *coli* IscS/Fdx [17]. The results presented here represent a single snapshot in a highly dynamic assembly process whose parts have not been fully identified.

The biogenesis of Fe-S clusters is a multistep process consisting of sulfur abstraction from cysteine, persulfide transfer, iron delivery and incorporation, and reductive generation of the Fe-S cluster, followed by transfer onto a target protein. Further studies will be necessary to identify any additional participants and determine how the biosynthetic steps are organized. In particular, the identity of the electron donor for Fe-S cluster biogenesis *in vivo* is unknown, and the role of SufB/SufC/SufD is yet to be determined.

The authors declare no financial conflict of interest.

## Supporting Information

S1 Fig Overlay of *BsSufS* with *E*. *coli* Cysteine Desulfurases. The structure of a *B*. *subtilis* SufS monomer (green) is superimposed with: **(A)** *E*. *coli* CsdA monomer (cyan; PDB ID 4LW2) with an r.m.s.d. of 3.30 Å over 401 Cα atoms; **(B)** *E*. *coli* SufS monomer (yellow; PDB ID 1I29) with an r.m.s.d. of 1.28 Å over 407 Cα atoms; and **(C)** *E*. *coli* IscS monomer (magenta; PDB ID 3LVL) with an r.m.s.d. of 5.55 Å over 389 Cα atoms
(TIFF)

S2 Fig Comparison of frataxin homologs. Frataxin usually consists of two α-helices and one 6-stranded β-sheet. Two additional helices appear in *Bs*Fra.
(TIFF)

S3 Fig Analysis of the *Bs*Fra/*Bs*SufU interaction by H/DX. Differences in H/D uptake of the interaction complex compared to each individual protein are mapped onto the structures of **(A)** *Bs*Fra (PDB ID 2OC6) and **(B)** *Bs*SufU (PDB ID 2AZH). The relative amount of deuterium incorporated is indicated by a color code ranging from blue (low; stable region) to red (high; flexible region). Black regions were not detected. N-terminal (NT) and C-terminal (CT).
(TIFF)

S4 Fig Analysis of the *Bs*Fra/*Bs*SufS interaction by H/DX. Differences in H/D uptake of the interaction complex compared to each protein alone are mapped onto the structures of **(A)** *Bs*Fra (PDB ID 2OC6), **(B)** the *Bs*SufS monomer, and **(C)** the *Bs*SufS homodimer. The relative amount of deuterium incorporated is indicated by a color code ranging from blue (low; stable region) to red (high; flexible region). Black regions were not detected. N-terminal (NT) and C-terminal (CT).
(TIFF)

S1 File H/DX Files. (XLS)
