# Supplementary figures and images for: Crystal Structure of Bacillus subtilis Cysteine Desulfurase SufS and Its Dynamic Interaction with Frataxin and Scaffold Protein SufU
Source: PLoS One. 2016 Jul 6;11(7):e0158749. doi: 10.1371/journal.pone.0158749 (PMC4934914; doi:10.1371/journal.pone.0158749)

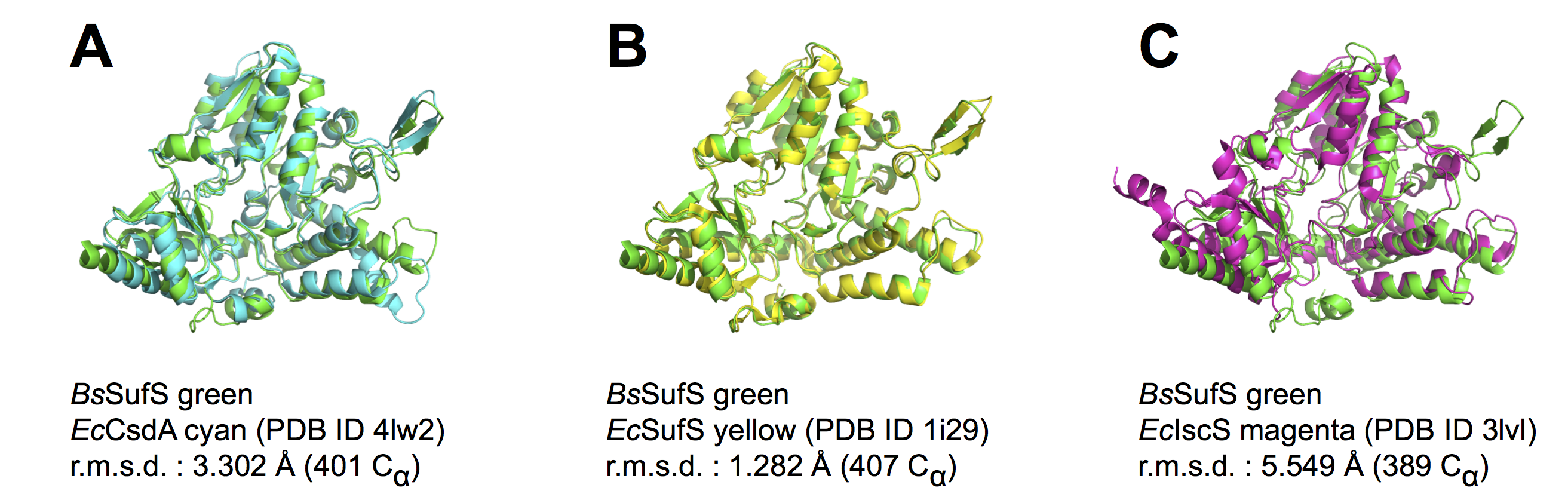

Supplement: S1 Fig — The structure of a B. subtilis SufS monomer (green) is superimposed with: (A) E. coli CsdA monomer (cyan; PDB ID 4LW2) with an r.m.s.d. of 3.30 Å over 401 Cα atoms; (B) E. coli SufS monomer (yellow; PDB ID 1I29) with an r.m.s.d. of 1.28 Å over 407 Cα atoms; and (C) E. coli IscS monomer (magenta; PDB ID 3LVL) with an r.m.s.d. of 5.55 Å over 389 Cα atoms (TIFF) [file pone.0158749.s001.tiff]

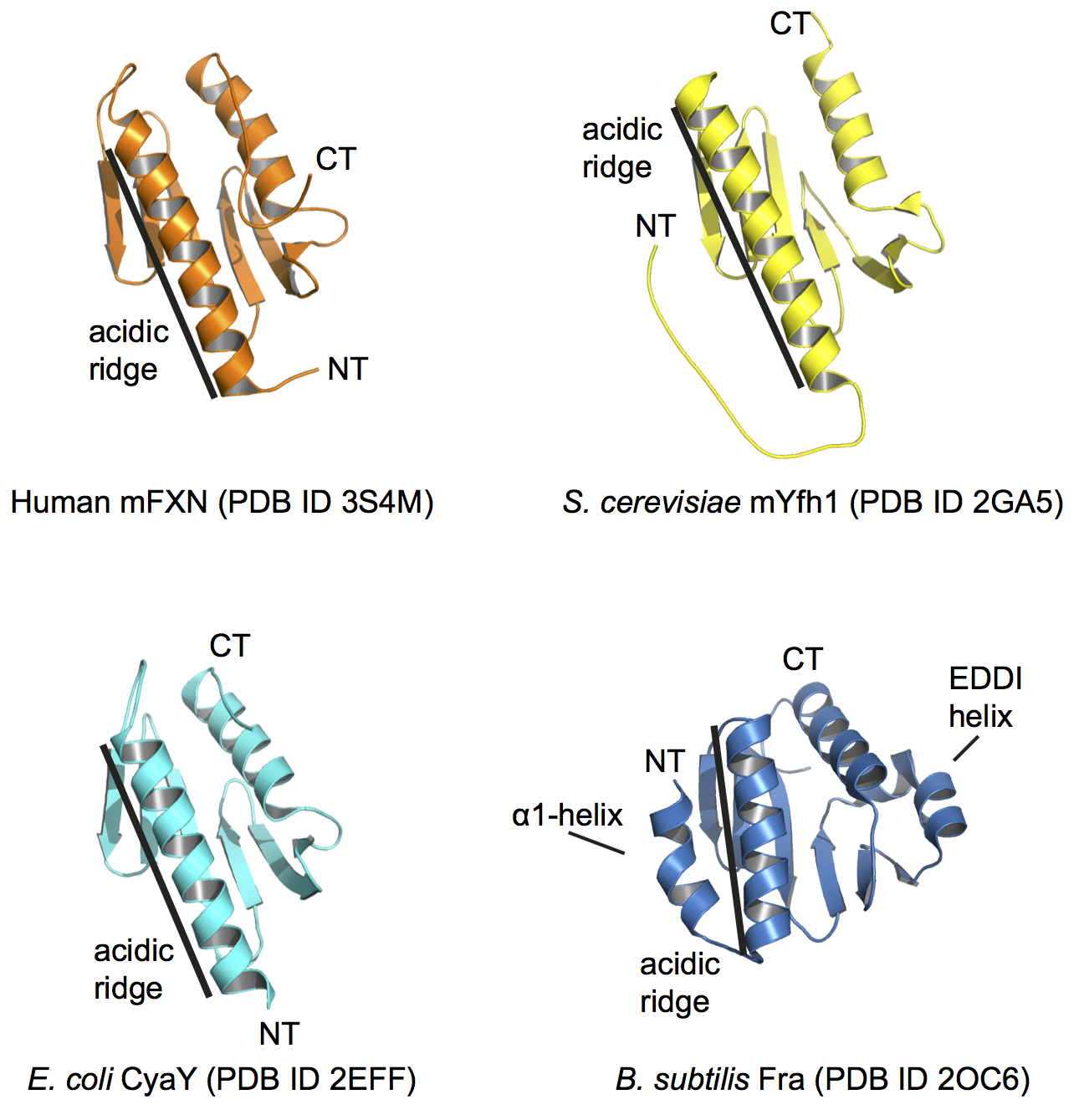

Supplement: S2 Fig — Frataxin usually consists of two α-helices and one 6-stranded β-sheet. Two additional helices appear in BsFra. (TIFF) [file pone.0158749.s002.tiff]

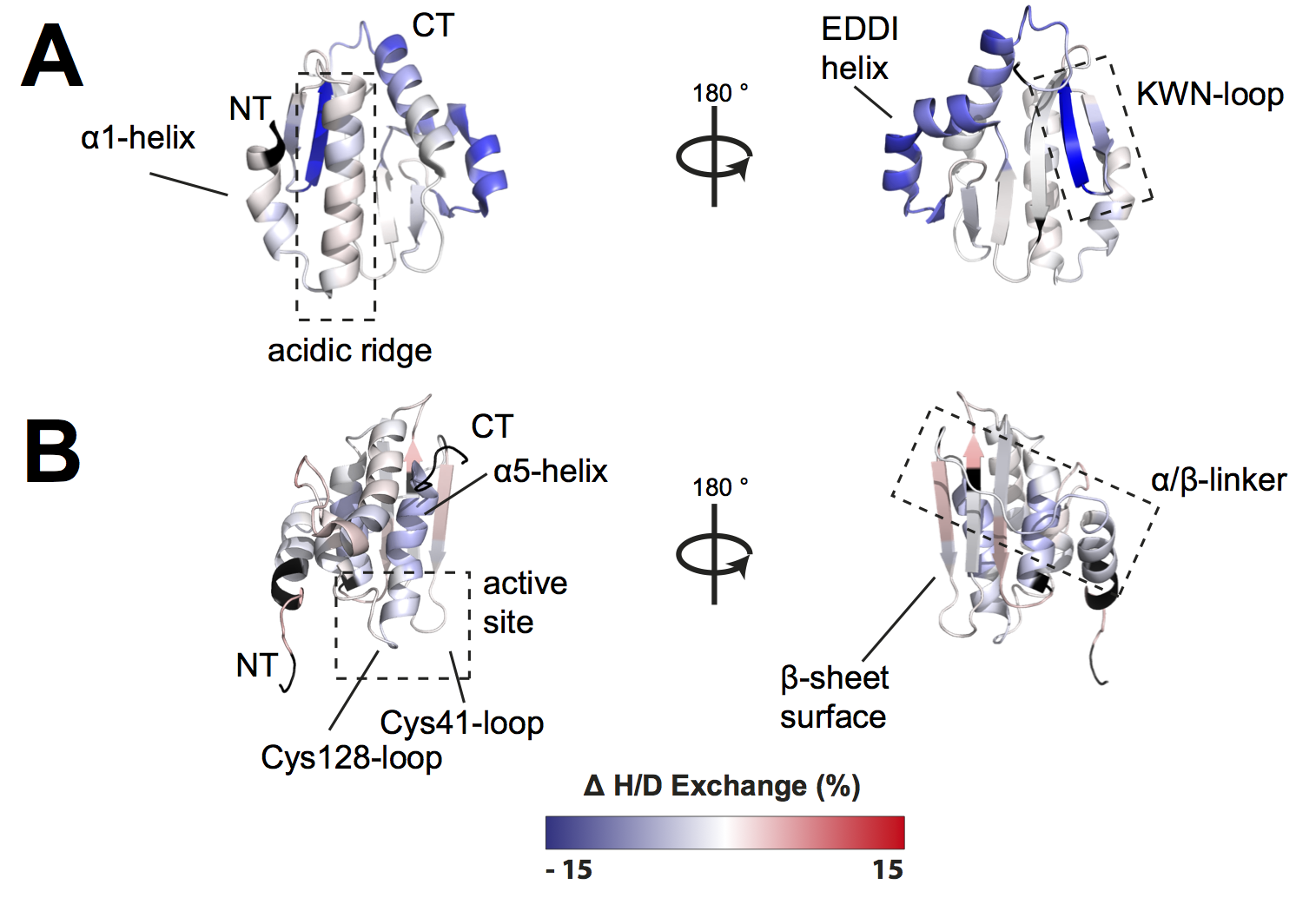

Supplement: S3 Fig — Differences in H/D uptake of the interaction complex compared to each individual protein are mapped onto the structures of (A) BsFra (PDB ID 2OC6) and (B) BsSufU (PDB ID 2AZH). The relative amount of deuterium incorporated is indicated by a color code ranging from blue (low; stable region) to red (high; flexible region). Black regions were not detected. N-terminal (NT) and C-terminal (CT). (TIFF) [file pone.0158749.s003.tiff]

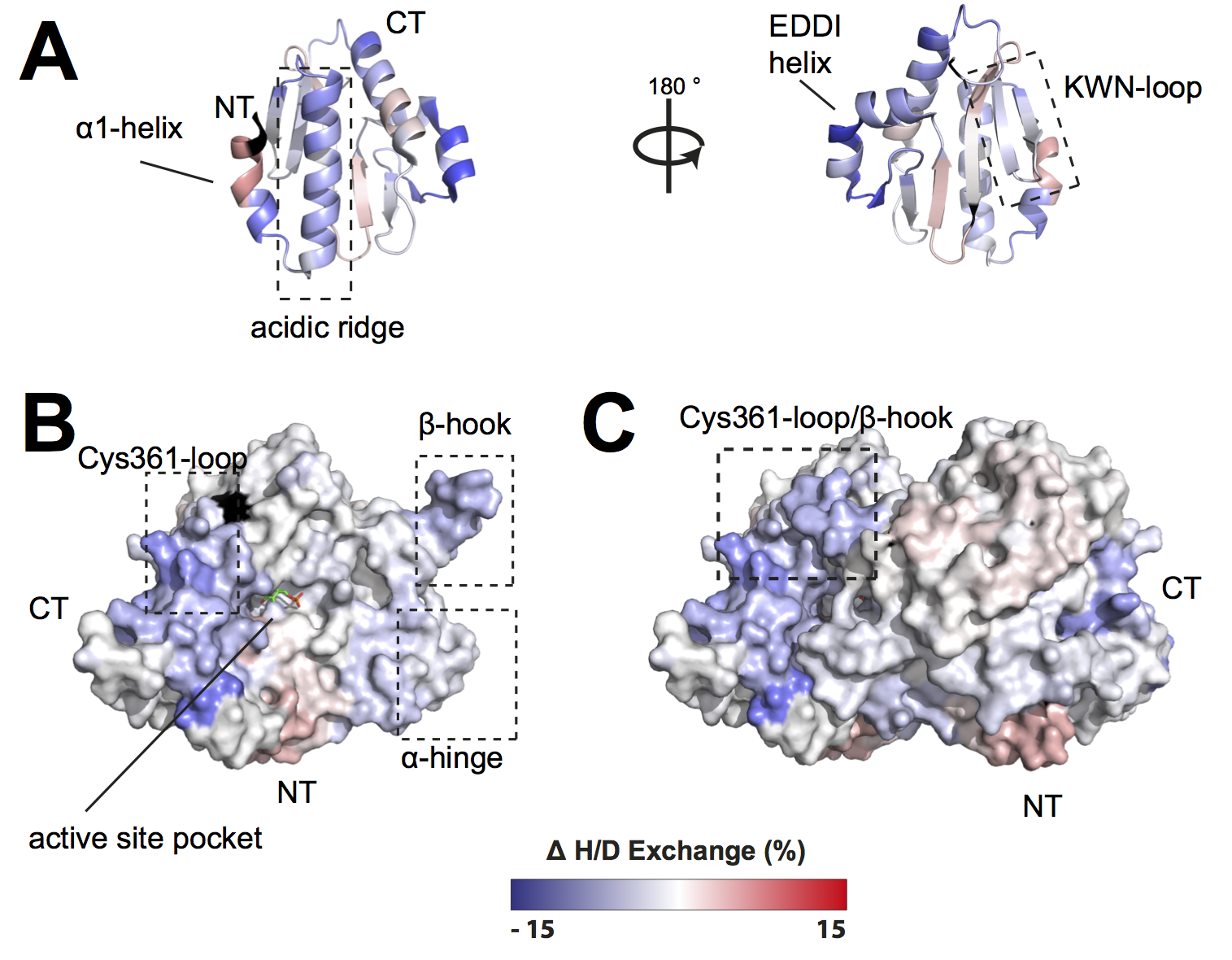

Supplement: S4 Fig — Differences in H/D uptake of the interaction complex compared to each protein alone are mapped onto the structures of (A) BsFra (PDB ID 2OC6), (B) the BsSufS monomer, and (C) the BsSufS homodimer. The relative amount of deuterium incorporated is indicated by a color code ranging from blue (low; stable region) to red (high; flexible region). Black regions were not detected. N-terminal (NT) and C-terminal (CT). (TIFF) [file pone.0158749.s004.tiff]
